# Green Tea Seed Isolated Theasaponin E1 Ameliorates AD Promoting Neurotoxic Pathogenesis by Attenuating Aβ Peptide Levels in SweAPP N2a Cells

**DOI:** 10.3390/molecules25102334

**Published:** 2020-05-16

**Authors:** Muhammad Imran Khan, Jin Hyuk Shin, Min Yong Kim, Tai Sun Shin, Jong Deog Kim

**Affiliations:** 1 Department of Biotechnology, Chonnam National University, San96-1, Dun-Duk Dong, Yeosu, Chonnam 550-749, Korea; imranbiotech1@gmail.com (M.I.K.); geobae@biolsystems.com (J.H.S.); 2 Department of Refrigeration Engineering, Chonnam Natational University, San96-1, Dun-Duk Dong, Yeosu, Chonnam 550-749, Korea; kmy@jnu.ac.kr; 3 Research center on Anti-Obesity and Health Care, Chonnam National University, San96-1, Dun-Duk Dong, Yosu, Chonnam 550-749, Korea; shints@jnu.ac.kr; 4 Department of Food Science and Nutrition, Chonnam National University, 77 Yongbong-ro, Buk-gu, Gwangju 550-757, Korea

**Keywords:** Alzheimer’s disease, amyloid precursor proteins, amyloidogenic pathway, α-secretase, SweAPP N2a cells, theasaponin E1

## Abstract

Alzheimer’s disease (AD) is the most frequent type of dementia affecting memory, thinking and behaviour. The major hallmark of the disease is pathological neurodegeneration due to abnormal aggregation of Amyloid beta (Aβ) peptides generated by β- and γ-secretases via amyloidogenic pathway. Purpose of the current study was to evaluate the effects of theasaponin E1 on the inhibition of Aβ producing β-, γ-secretases (BACE1, PS1 and NCT) and acetylcholinesterase and activation of the non-amyloidogenic APP processing α-secretase (ADAM10). Additionally, theasaponin E1 effects on Aβ degrading and clearing proteins neprilysin and insulin degrading enzyme (IDE). The effect of theasaponin E1 on these crucial enzymes was investigated by RT-PCR, ELISA, western blotting and fluorometric assays using mouse neuroblastoma cells (SweAPP N2a). theasaponin E1 was extracted and purified from green tea seed extract via HPLC, and N2a cells were treated with different concentrations for 24 h. Gene and protein expression in the cells were measured to determine the effects of activation and/or inhibition of theasaponin E1 on β- and γ-secretases, neprilysin and IDE. Results demonstrated that theasaponin E1 significantly reduced Aβ concentration by activation of the α-secretase and neprilysin. The activities of β- and γ-secretase were reduced in a dose-dependent manner due to downregulation of *BACE1*, presenilin, and nicastrin. Similarly, theasaponin E1 significantly reduced the activity of acetylcholinesterase. Overall, from the results it is concluded that green tea seed extracted saponin E1 possess therapeutic significance as a neuroprotective natural product recommended for the treatment of Alzheimer’s disease.

## 1. Introduction

Alzheimer’s disease (AD) is a genetically complex and progressive neurodegenerative disorder. The incidence of AD is rapidly increasing in developed countries, and the associated dementia is one of the greatest healthcare challenges affecting people worldwide [1]. Causes of AD development and pathogenicity may be environmental or genetic and symptoms vary between individuals according to the severity of the disease. General symptoms in the initial stages usually manifest before the age of 65 and involve memory loss, confusion, speech problems, and delusions [2]. As the disease worsens, the individual may lose the ability to focus, communicate, and recognize people or places, and may also have difficulty in walking and swallowing. 

There are several causes of AD at molecular level, collectively leading to synaptic injury, neurodegeneration, and eventually neuronal loss [3,4]. The five established hypotheses are: amyloid isoprenoid change hypothesis, tau hypothesis, cholinergic hypothesis, calcium hypothesis and the amyloid-beta (Aβ) hypothesis [5,6,7,8]. However, the two main hypotheses are the Aβ proteins and hyperphosphorylated tau, which make neurofibrillary tangles and senile plaques causing microglial cell proliferation and neurodegeneration [9]. The pathogenesis of AD is believed to be caused by progressive accumulation of Aβ proteins resulting from the amyloidogenic cleavage of Aβ precursor protein (APP) by β- and γ-secretases to form Aβ40, Aβ42 and Aβ43 [10,11]. β-Secretase cleaves APP at Met671 and Asp672 to generate Aβ40 and Aβ42 [12]. Aβ40 is comparatively nontoxic in normal quantities and is found in the brain of normal aged people [13]. Similarly, γ-secretase cleaves APP at Ile712, Thr714 or Val715 to produce Aβ40, Aβ42 and Aβ43 [14,15]. The fragments Aβ42 and Aβ43 are neurotoxic and are the primary components of plaques in the brains of AD patients [16]. The imbalance between production and clearance of different Aβ proteins leads to aggregation and accumulation. Hence, enhancement of β- and γ-secretase activity is responsible for production and accumulation of the neurotoxic fragments Aβ42 and Aβ43, leading to the formation of plaques and progressive neurodegeneration eventually causing AD. 

α-Secretase is a disintegrin and metalloproteinase (ADAM) that cleaves APP in the non-amyloidogenic pathway and generates neuroprotein fragments. ADAM10 is the principal metalloproteinase encoded by the *ADAM10* gene and is part of the α-secretase family [17]. Β-site amyloid precursor protein cleaving protease enzyme (BACE) is a β-secretase that cleaves APP at the β-site via the amyloidogenic pathway and generates neurotoxic Aβ. BACE1 is the principal β-secretase controlled by the *BACE1* gene [18]. γ-secretase is a multi-subunit protease complex generating Aβ peptides via proteolytic processing of APP through the amyloidogenic pathway. Nicastrin (NCT) and presenilin (PS), a multipass transmembrane protein, are crucial in the catalytic function of γ-secretase [19].

The most effective approach to treating AD is reducing Aβ production, which is achieved by activating α-secretase and inhibiting β- and γ-secretases, increasing the expression of the proteolytic enzymes neprilysin, insulin-degrading enzymes (IDE), and apolipoprotein E (apoE), which are crucial for Aβ degradation and clearance [20,21], and activating the lysosomal and non-lysosomal pathways that are involved in Aβ degradation and clearance [22,23]. Aβ oligomers and fibrils are made from abnormal progression and accumulation of Aß resulting in the formation of plaques that cause neuronal toxicity, synaptic loss and, eventually, neuronal degradation [24,25,26].

There have been considerable advances in identifying and revealing the genes involved in the development of AD. Genes currently known to be involved in the development of AD are presenilin and nicastrin (*PS1, CNT*) (γ-secretase), *BACE1* (β-secretase), *APP*, and *ApoE* [27]. APP and PS1 function in a single pathway, the APP processing, centrally involves in AD pathogenesis usually the case of familial AD. In sporadic AD alteration of *ApoE* gene is the main risk factor and ε4 allele of APOE gene is highly frequent in late-onset AD (LOAD). Another important factor in AD patients is cholinergic dysfunction due to the reduced amount of the neurotransmitter acetylcholine (ACh) in the brain. ACh is crucial for cholinergic nerve signal transmission and imbalance of ACh in the synaptic cleft leads to impaired neuronal transmission, impaired function, and memory deficits [28]. Acetylcholinesterase (AChE) is responsible for hydrolysis of ACh, which is a normal physiological process; however, the altered or elevated function of AChE causes reduction in ACh and affects the neuronal signal transmission processes. Aβ amyloid proteins that make up the senile plaques interact with ACh receptors (nAChRs) in the brain and induce neuronal apoptosis, which affects learning and memory ability [29]. In animal models, especially Aβ-infused rats, it has been shown that Aβ amyloids cause inactivation of the nAChR α7, leading to long-term impairment [30].

Saponins are naturally-occurring compounds with a diverse range of biological effects and medicinal values. Green tea saponins have been reported to have many biological effects, including antimicrobial, anti-cancer, adjuvant and gastroprotective properties [31]. The biological activities of saponins depend on their chemical structures and are affected by factors such as the saponin nucleus type, number of sugar chains, and types of functional substituents [32]. Saponins have therapeutic effects owing to their chemical structures and can interact in various molecular pathways. However, previous pharmacological studies are limited and green tea saponins have not been reported previously for anti-AD and neuroprotective effects. The goal of this study was to evaluate the therapeutic potential of theasaponin E1 on the reduction of Aβ amyloids by regulating the associated signalling molecules and enzymes. Our results showed that theasaponin E1 has significantly reduced Aβ in SweAPP N2a cells by reducing its production through inhibition of amyloidogenic cleavage of APP by β-secretase, γ-secretase etc. 

## 2. Results

### 2.1. Saponin Extraction and Analysis

Saponins were extracted from green tea seed and in the saponins mixture following major saponins were detected by LC/TOF/MS: theasaponin E1, theasaponin C, assamsaponin A (C_57_H_88_O_25_), theasaponin E3 (C_57_H_88_O_26_), theasaponin A1 (C_57_H_90_O_26_), assamsaponin B (C_61_H_92_O_28_) and theasaponin A3 (C_61_H_94_O_28_). 

**Figure 1:**
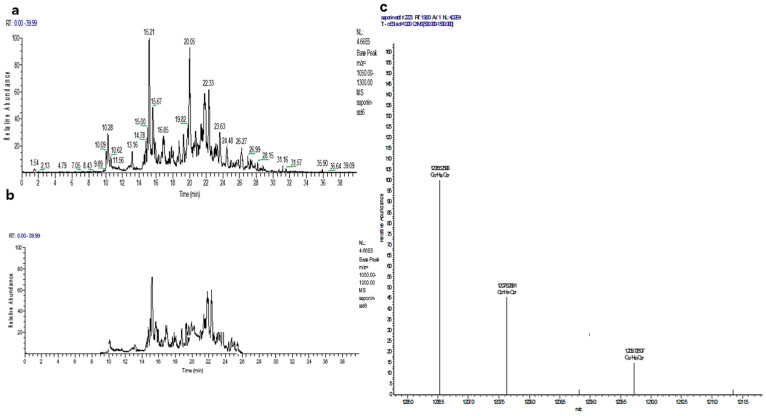
LC/TOF/MS analysis of *Camellia sinensis* seed extracted saponin mixture and the isolated pure saponins fraction (Base peak intensity chromatogram at *m*/*z* 1050–1300). (**a**) LC-MS chromatogram of the saponin mixture showed the presence of theasaponin E1, theasaponin C, assamsaponin A (C_57_H_88_O_25_), theasaponin E3 (C_57_H_88_O_26_), theasaponin A1 (C_57_H_90_O_26_), assamsaponin B (C_61_H_92_O_28_) and theasaponin A3, (C_61_H_94_O_28_). (**b**) LC-MS chromatogram of the isolated theasaponin E1 reached fraction. (**c**) LC-MS/MS spectra of the isolated saponin fraction showed theasaponin E1.

NMR was performed for structure characterization of the saponins. The pure saponin fraction obtained from the saponin mixture by preparative HPLC contained mainly theasaponin E1, along with a very small amount of theasaponin E3. Theasaponin E1 was further used for experiments in this study. LC/TOF/MS of the saponin mixture and pure isolated theasaponin E1 is presented in Figure 1. NMR data are provided in the Supplementary File (S1).

### 2.2. TLC Analysis

**Figure 2:**
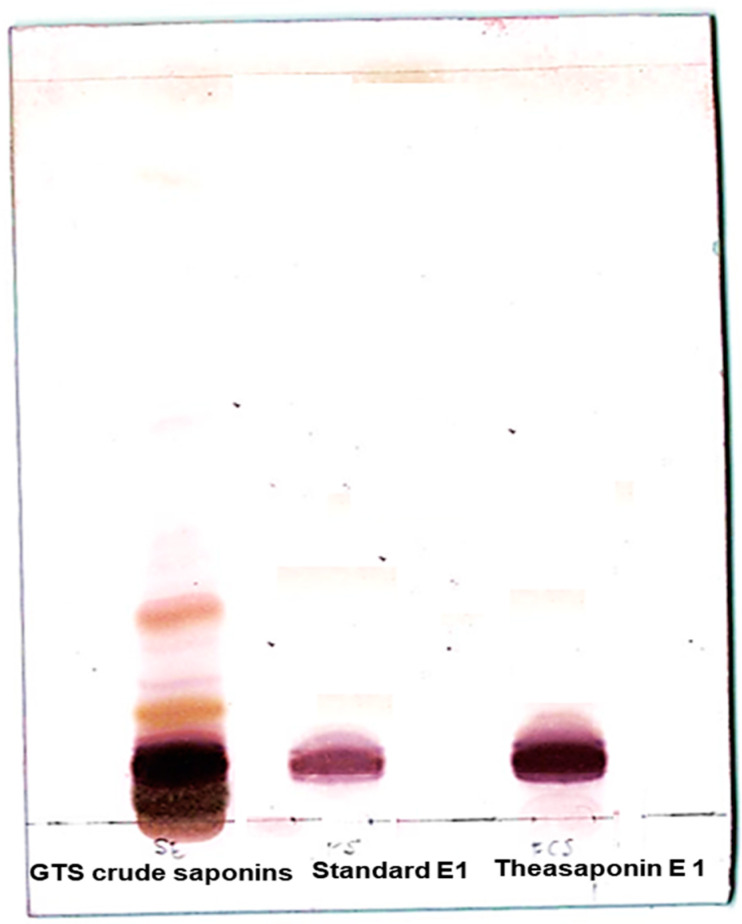
Thin layer chromatography (TLC) of the green tea seed extracted saponin mixture and the purified isolated theasaponin E1 in comparison with the commercial grade standard theasaponin E1.

The mixed saponin fraction and the purified saponin fraction were analysed by thin-layer chromatography (TLC). The purified saponin E1 was identified by comparing the TLC band with commercial grade standard theasaponin E1. Results confirm the presence of theasaponin E1 in the isolated fractions (Figure 2).

### 2.3. MTT Assay 

**Figure 3:**
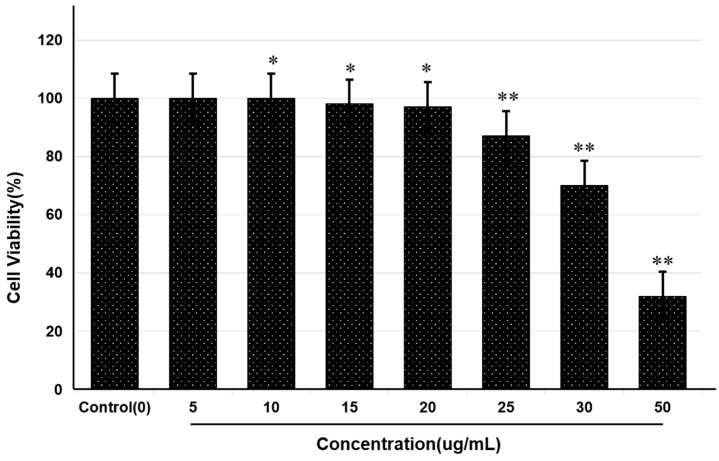
Toxicity determination of theasaponin E1 on mouse neuroblastoma cells by MTT assay. Percentage cell viability was determined for control and various concentrations of theasaponin E1. Experiments were performed in triplicate. Data are the mean ± SEM. Data are statistically significant at *p* < 0.05. (* represents significance at *p* < 0.05, ** represents significance at *p* < 0.01 to control group).

The toxicity of saponins to mouse blastoma cells was determined by MTT assay, and the safe level of saponins was determined by the percentage of viable cells. Cells not treated with saponins were used as the negative control and had 100% viability. The results showed that treatment with 20 μg/mL of pure saponin was nontoxic, with greater than 90% viability (Figure 3). Increasing the concentration beyond this level led to decreased cell viability and increased toxic effects.

### 2.4. Acetylcholinesterase Inhibitory Assay

**Figure 4:**
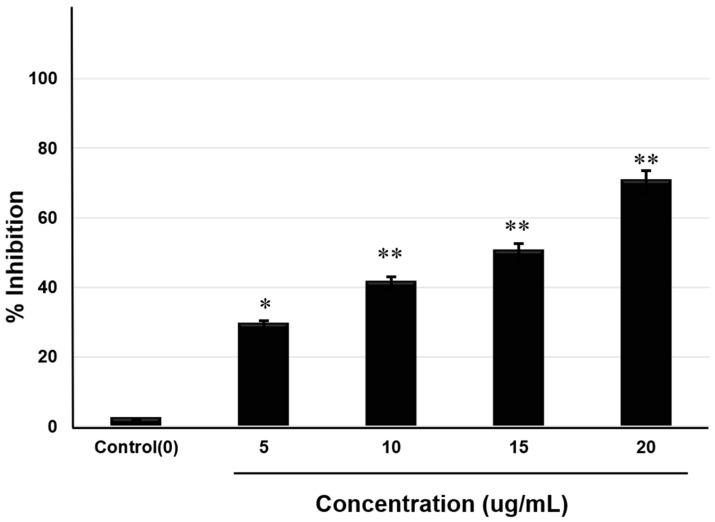
Acetylcholinesterase inhibitory activity of theasaponin E1. Percent inhibition was measured for each concentration and control according to the manufacturer’s protocol. Data are expressed as mean ± SEM. Data were analysed with one-way ANOVA (*n* =  3). Data are statistically significant at *p* < 0.05. (* represents significance at *p* < 0.05, ** represents significance at *p* < 0.01).

AChE is an enzyme that hydrolyses the neurotransmitter ACh to acetate and choline. AChE inhibitory activity assay was conducted using an AChE activity assay kit. This assay is an optimized version of the Ellman’s method in which thiocholine, produced by AChE, reacts with 5,5′- dithiobis-(2-nitrobenzoic acid) (DTNB) to form a colorimetric (412 nm) product proportional to the AChE activity. Activity of the enzyme was calculated for each group (control and saponin-treated) according to the kit instructions. Results showed that saponin treatment significantly reduced the activity of AChE in a dose-dependent manner. The enzyme showed highest activity in the untreated control group, whereas the lowest activity occurred in the cells treated with the highest concentration of saponins (20 μg/mL) (Figure 4). 

### 3.5. Effects of Saponins on the Activities of α-Secretase (ADAM10), β-Secretase (BACE1), γ-Secretases (PS1 and NCT), IDE and Neprilysin 

**Figure 5:**
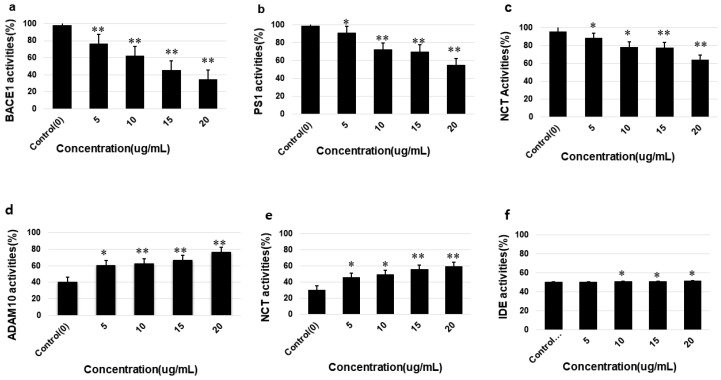
Effects of theasaponin E1 on the activities of APP cleaving proteases and Aβ degrading enzymes in vitro determined by fluorometric assays. Percent activity was measured for control and treatment groups for each enzyme. (**a**) Effects of theasaponin E1 on β-secretase (BACE1) activities. (**b**) Effects of theasaponin E1 on γ-secretase (PS1) activities. (**c**) Effects of theasaponin E1 on γ-secretase (NCT). (**d**) Effects of Theasaponin E1 on α-secretase (ADAM10) activities. (**e**) Effects of theasaponin E1 on neprilysin activities. (**f**) Effects of theasaponin E1 on insulin degrading enzyme (IDE) activities. Data are expressed as mean ± SEM. Data were analysed with one-way ANOVA (*n* =  3). (*p < 0.05; ** *p* < 0.01).

To investigate the effects of isolated saponins on the activation or inhibition of α-secretase (ADAM10), β-secretase (BACE1), γ-secretases (PS1 and NCT), IDE and neprilysin, and the activity of these enzymes was measured by fluorometric assays using enzyme-specific kits. SweAPP N2a cells were treated with various nontoxic concentrations of the isolated theasaponin E1 and cell lysate was prepared. The assays were performed according to the kit instructions and percent activity was calculated for each enzyme based on the untreated control cells. Results show that treatment with saponins increased α-secretase and neprilysin activity but did not change the IDE activity. Theasaponin E1 showed a higher activation effect on α-secretase as compared to neprilysin. In addition, the activity of β-secretases and γ-secretase was found to be significantly decreased with increasing concentrations of saponin treatment; however, the inhibitory effect was comparatively higher for β -secretases. Inhibitory effects of theasaponin E1 was almost similar for both the subunits PS1 and NCT, etc. (Figure 5). 

### 3.6. RT-PCR Analysis

**Figure 6:**
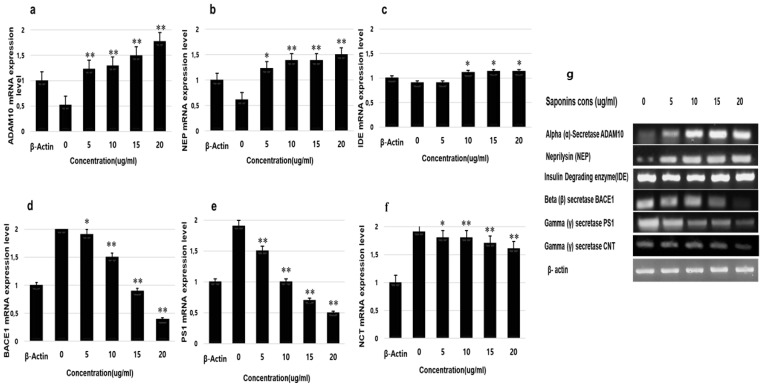
Effects of theasaponin E1 on mRNA expression levels of APP cleaving proteases and Aβ degrading enzymes in vitro determined by RT-PCR. Relative mRNA expression levels of genes of various enzymes under the effects of theasaponin E1. (**a**) Relative mRNA expression level of α-secretase (ADAM10). (**b**) Relative mRNA expression level of neprilysin. (**c**) Relative mRNA expression level of insulin degrading enzyme (IDE). (**d**) Relative mRNA expression level of β-secretase (BACE1). (**e**) Relative mRNA expression level of γ-secretase (PS1). (**f**) Relative mRNA expression level of γ-secretase NCT. (**g**) Expression level of *ADAM10, BACE1, PS1, NCT, neprilysin* and *IDE* genes. Visualized the PCR products on agarose gel. Data are expressed as mean ± SEM. Data were analysed with one-way ANOVA (*n* = 3). Data are statistically significant at *p* < 0.05. (* *p* < 0.05; ** *p* < 0.01 to control group).

RT-PCR was used to measure the expression of genes involved in the Aβ pathway using gene-specific primers (Table 1). The results revealed a significant effect of saponin treatment on the expression of α-secretase (ADAM10) and neprilysin. The expression level of ADAM10 and neprilysin was upregulated in a dose-dependent manner. Saponin treatment did not significantly affect the expression of IDE. Saponin treatment significantly decreased the expression of the β-secretase (BACE1) and the γ-secretase (PS1), with a greater suppression of BACE1 than PS1. theasaponin E1 showed comparatively lower suppressive effects to γ-secretase subunit NCT as compared to PS1. Relative expression levels of ADAM10, BACE1, PS1, NCT, neprilysin and IDE were calculated and compared to that of ß-actin control (Figure 6).

### 3.7. Aß Peptides and APP Analysis by ELISA 

**Figure 7:**
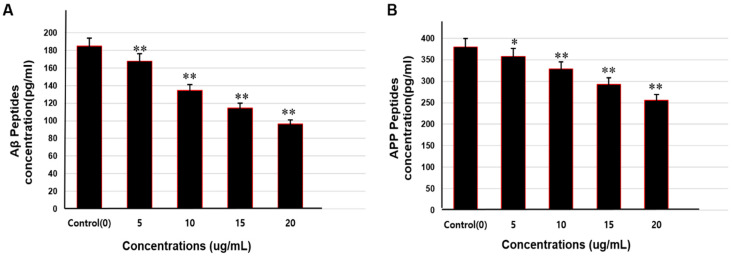
Effect of theasaponin E1 on the level of Aβ peptides and APP level quantified by ELISA (due to enhancement or reduction in the activities of APP processing and Aβ amyloid producing or degrading enzymes). (**A**) Aβ quantification by ELISA after treatment of the cells with various concentrations of E1 theasaponin. (**B**) APP quantification by ELISA after treatment of the cells with various concentration of E1 theasaponin. Data are expressed as mean ± SEM. Data were analysed with one-way ANOVA (*n*  =  3). Data are statistically significant at *p* < 0.05. (* *p* < 0.05; ** *p* < 0.01 to control group).

The level of Aβ peptides and APP was measured to determine the inhibitory or activating effects of saponins on the APP-processing proteolytic secretases and Aβ-degrading enzymes. SweAPP N2a cells were treated with various nontoxic concentrations of the isolated saponins and cell lysate was prepared for ELISA experiments. The results show that the level of Aβ peptides and APP was significantly reduced as compared to control in a dose-dependent manner (Figure 7).

### 3.8. Western Blotting

**Figure 8:**
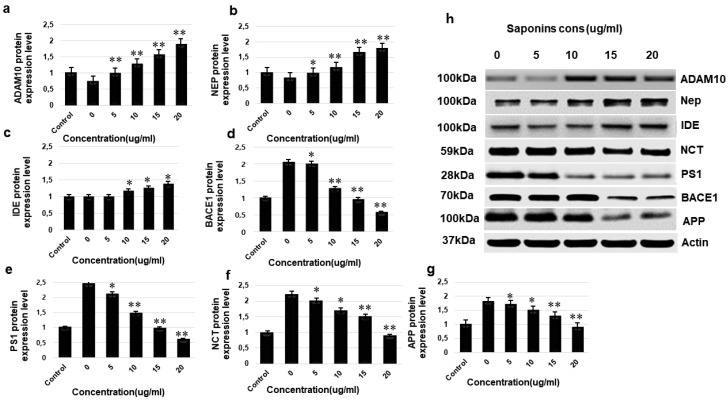
Effects of theasaponin E1 on the protein expression level of *ADAM10, BACE1, PS1, NCT, neprlysin, IDE* and *APP* determined by western blotting. Relative proteins expression levels of various proteins normalized (fold change) to control (β-actin). (**a**) Relative proteins expression level of α-secretase (ADAM10). (**b**) Relative proteins expression level of neprilysin. (**c**) Relative proteins expression level of insulin degrading enzyme (IDE). (**d**) Relative proteins expression level of β-secretase (*BACE1*). (**e**) Relative proteins expression level of γ-secretase presenilin 1 (*PS1*). (**f**) Relative proteins expression level of γ-secretase nicastrin *NCT*. (**g**) Relative expression level of *APP*. (**h**) Expression of APP, ADAM10, BACE1, PS1, NCT, neprilysin and IDE proteins, visualized bands on membrane. Data are expressed as mean ± SEM. Data were analysed with one-way ANOVA (*n* = 3). Data are statistically significant at *p* < 0.05. (*p < 0.05; ** *p* < 0.01). Data are expressed as mean ± SEM.

Western blotting was performed to determine the effects of the green tea seed extracted theasaponin E1 on the protein expression of *ADAM10, BACE1, PS1, NCT, IDE, neprilysin, APP* and Aβ peptides. Antibodies used in Western blotting to target these proteins are listed in Table 2. The expression level of ADAM10 and neprilysin increased significantly in a dose-dependent manner whereas the expression of IDE did not change. Saponin treatment decreased the expression of BACE1 (β-secretase) and the catalytic subunit of γ-secretase (PS1), but the expression of the other subunit NCT was not affected. Saponin treatment suppressed expression of BACE1 comparatively higher than PS1. There was a significant decrease found in APP and Aβ level. Relative expression level clearly confirmed the activation of ADAM10 and neprilysin and the significant inhibition of BACE1 and PS1 compared to the control (Figure 8). 

## 3. Discussion

Alzheimer’s disease is the most common type of dementia affecting people worldwide. The major hallmark of AD is pathological neurodegeneration due to the formation of extracellular senile Aβ plaques and intraneuronal neurofibrillary tangles aggregates of hyperphosphorylated tau [33]. The major component of the senile plaque are Aβ peptides, which are produced and accumulated in the brain parenchyma and cerebral blood vessels. Aβ is generated from cleavage of APP by β-secretase and γ-secretase through the amyloidogenic pathway. α-Secretase normally cleaves and processes APP by the non-amyloidogenic pathway and does not create the toxic Aβ peptides [34]. The enzyme acetylcholinesterase is also responsible for neurodegeneration due to degradation of the neurotransmitter acetylcholine and elevation of Aβ levels. Neprilysin and insulin-degrading enzyme play a role in degradation and clearance of the Aβ peptides and there are evidences that AD caused due to failure in the function or deficiencies of these proteins resulting in elevated Aβ level [35]. Hence, all these proteins are therapeutic targets for reducing or preventing neurodegeneration and treating Alzheimer’s disease. Several studies suggest that reduction of the amount of Aβ peptides in the brain, either by directly reducing their production and aggregation or by promoting their degradation and clearance, could attenuate AD pathology [36]. Aβ peptides are formed after sequential cleavage of the Aβ precursor protein by β-secretase and γ-secretase [37]. The β-secretase BACE1 can cleave several substrates crucial in neuronal function, including APP, by amyloidogenic processing [38,39,40]. γ-Secretase is a complex protease consisting of at least four proteins, including the catalytic subunit presenilin-1, and NCT, which functions in the proteolysis of APP and other proteins and releases peptide fragments with important biological functions [41,42]. Similarly, acetylcholinesterase is found to be a progressive cause of AD because of its continuous degradation of the neurotransmitter acetylcholine. In Alzheimer’s patients, α7 nAChR, the most abundant subtype receptor of acetylcholine in brain, is significantly reduced due to Aβ amyloid production. Hence Aβ generation, α, β, and γ-secretases, neprilysin and acetylcholinesterase are crucial molecular targets for AD therapeutic drug screening. SweAPP N2a cells as in vitro modules are a good choice for investigating the therapeutic effects of drugs on modulation of app processing and Aβ induced AD pathogenesis however, this model limits the studies of other hypothesis and molecular targets responsible for AD pathogenesis. 

The green tea plant *(Camellia sinensis)* is enriched in several bioactive compounds, including saponins, with crucial biological and pharmacological activities and has been utilized in nutraceutical, pharmaceutical and cosmetics industries through various formulations. In the current study, we extracted and isolated pure theasaponin E1 from green tea seed and evaluated its effects on the reduction or inhibition of Aβ amyloid, the major cause of neurodegeneration in Alzheimer’s disease. Our results demonstrated that theasaponin E1 significantly reduced Aβ amyloid by activating APP processing by the non-amyloidogenic pathway via enhancing ADAM10 activities and reduced the amyloidogenic cleavage of APP by inhibiting BACE1 and PS1. In addition, theasaponin E1 enhanced the activity of neprilysin, which caused reduction in Aβ amyloid. Theasaponin E1 inhibited amyloidogenic Aβ production by suppressing BACE1 and PS1 protein expression and activity. We found that theasaponin E1 suppressed the expression level of *BACE1, PS1* and *APP* and enhanced the expression level of *ADAM10* and *neprilysin* in a dose-dependent manner but did not alter the expression of IDE and also comparatively less effective to *NCT*. Aβ reduction might be due to the enhanced non-amyloidogenic APP processing by ADAM10 and decreased amyloidogenic processing by BACE1 and PS1, supported by the reduced expression of APP. Additioanlly, the enhancement of neprilysin activity and expression by theasaponin E1 might contribute to the reduction of Aβ level. The overall results revealed that theasaponin E1 inhibited Aβ production in association with suppression of the amyloidogenic pathway and activation of the non-amyloidogenic pathway and its degradation by the degrading enzymes. Inhibition or Reduction in Aβ production is crucial for preventing or reducing neurodegeneration and AD. Natural products with the potential of attenuating Aβ amyloid are vital products for the prevention or treatment of AD. Green tea possess a number of bioactive compounds with profound medicinal and pharmacological values. Green tea has been reported for anticancer, antidiabetic, anti-obesity, antiangiogenic and antimicrobial activities [43,44,45,46] Green tea has therapeutic effects on human brain function, ameliorates liver injury and enhances immunity. [47,48]. In the present study we proved that green tea seed isolated thaesaponin E1 has significant potential of mitigating the neurotoxic Aβ amyloid. 

## 4. Materials and Methods

### 4.1. Extraction of Saponins

Green tea (*Camellia sinensis (L.) O. Kuntze*) seeds were collected in natural conditions from Myeongin Shin Kwang-Soo’s tea farm Suncheon (34.9506° N, 127.4872° E) Jeonnam Chonnam, Republic of Korea. The plant was identified, and a voucher herbarium specimen was deposited to the National Agrobiodiversity Center (Wanju-gun, Korea). A voucher number (HCCN60938) was obtained for the specimen. Pure saponins were extracted from green tea (*Camellia sinensis (L.) O. Kuntze*) seeds (GTS) as previously described with some improvement [43,44]. Briefly, GTS were ground into fine powder and defatted with n-hexane by sonication at 30 °C for 6 h and then dried. The resulting dried defatted green tea seed powder was then extracted with 70% EtOH at 60 °C for 8 h by continuous reflux. The extract was filtered and concentrated with a rotatory vacuum evaporator. The extract was further treated with butanol and water mixture and again concentrated with a rotary rotatory evaporator. This crude extract was then passed through a nonpolar macroporous resin (D101). The extract (50  g) dissolved in 100 mL double distilled water was passed through the resin column and eluted with 0.4 N NaOH, followed by neutralization of the extract and resin mixture with HCl and elution with 100% EtOH. In this process, unwanted colour compounds were separated and discarded, and the brownish saponin-containing fraction was retained. Further fractionation and purification of the crude saponins were performed by column chromatography using a C-18 Luna column. Crude saponins (100 g) were dissolved in 100 mL 100% MeOH and loaded onto the C-18 Luna column. Elution was started with 300 mL 10% MeOH followed by 200 mL 60% MeOH and the saponin fraction was eluted finally with 300 mL 100% MeOH. The obtained saponin-rich fraction was again subjected to elution with 100% MeOH on the C-18 Luna column to obtain a fraction enriched with pure theasaponin E1. This fraction was further subjected to preparative HPLC (Develosil ODS-HG-5, MeCN-0.05% aqueous TFA 55:45, 4 mL/min) for isolation of the pure theasaponin E1. The purified isolated saponin was identified by thin layer chromatography (TLC), liquid chromatography time-of-flight mass spectrometry (LC/TOF/MS) and NMR. The resulting fraction was dried with lyophilization to form a yellow powder and aqueous extract in double distal water was used for experiments.

### 4.2. Thin-Layer Chromatography

The isolated rheasaponin E1 was identified by thin-layer chromatography (TLC) using a standard theasaponin E1 for comparison. The saponin samples (10 mg/mL) were dissolved in 80% EtOH and applied to a normal-phase TLC plate (TLC silica gel 60, glass plates 10 × 20 cm, Merck, Darmstadt, Germany). The TLC plate was then kept in the development chamber containing n-butanol, water, and acetic (84:14:17) as the development solution. After the saponins developed on the plates, it was dried and then sprayed with 15% H_2_SO_4_. The plate was kept at 115 °C for 15 min and the saponin bands were visualized.

### 4.3. MTT Assay

N2a mouse neuroblastoma cells overexpressing the Swedish mutant APP (SweAPP N2a) were used in the present study with 10% FBS. Toxicity of the pure saponins to SweAPP N2a cells was determined by MTT assay. Cells were seeded in 96-well plate at a density of 5 × 10^3^ cells/well and were incubated in a humidified 5% CO_2_ incubator at 37 °C for 24 h. Cells were then treated with various concentrations (5, 10, 15, 20, 25 and 30 μg/mL) of pure saponins for 24 h. Untreated cells were used as the control. After incubation, 0.5% MTT solution was added to each well of the plate containing cells and incubated for 4 h. After and the incubation, the media was aspirated from each well and dimethyl sulfoxide was added to dissolve formazan crystals and produce the purple colour. Absorbance of the 96-well plate was measured at 540 nm using a microplate reader. Percentage cell viability was calculated in comparison to the control and the concentration of saponin for which more than 90% of the cells remained viable was selected for further use in this study. Each experiment was repeated in duplicate.

### 4.4. Acetylcholinesterase Activity Inhibitory Assay

Inhibition of acetylcholinesterase activity was determined using Ellman’s colorimetric method as modified by Eldeen et al. [49]. The activity was determined by using an acetylcholinesterase activity assay kit (Sigma Aldrich) according to the manufacturer’s instructions. Briefly, the working reagent was prepared freshly as 2 mg/200 µL of assay buffer provided in the kit and 190 µL per well was transferred to a 96-well plate. Each concentration of the saponin (10 µL) was then added to the respective wells of the plate and was mixed thoroughly. Wells containing only distilled water (200 µL) were used as the blank. The calibrator (200 µL) was added to separate wells. Plates were then incubated at room temperature. After 2 min, the initial measurement was done by measuring the absorbance at 412 nm and after 10 min the final absorbance was measured at 412 nm. Inhibition and activity were calculated according to the formulas provided by the manufacturer.

### 4.5. Enzyme Activity Analysis by Fluorometric Methods

Enzyme activities were measured using the SensoLyte® 520 kit specific for each enzyme following the manufacturer’s instructions. SweAPP N2a cells were cultured in six-well plates incubated at 37 °C in a 5% CO_2_ incubator. After attachment, cells were treated with various nontoxic concentrations of the purified saponins. Untreated cells were used as the control. Cell lysate was prepared and used in each enzyme-specific assay. Absorbance was measured at 450 nm using an ELISA microplate reader. Data were analysed and compared following the manufacturer’s instructions. Each experiment was repeated in triplicate. 

### 4.6. RT- PCR Analysis

SweAPP N2a cells were cultured in DMEM media supplemented with 10% FBS in six-well plates and were incubated at 37 °C in a CO_2_ incubator. These cells were then treated with different concentrations of pure saponins and incubated for 24 h. Total RNA was extracted from cells using an RNA isolation kit (Sigma Aldrich, Cat. No. 83913) followed by DNase treatment. Quantification of the isolated RNA was performed on a NanoDrop 2000/2000c spectrophotometer (Thermo Fisher Scientific, Wilmington, DE, USA). The RNA (500 ng) was then reverse transcribed to cDNA with Revert Aid Premium First Strand cDNA Synthesis Kit (Thermo Fisher Scientific No. K1621). cDNA was quantified with the Nano Drop spectrophotometer and processed for real-time qPCR with gene-specific using a Thermo Scientific qPCR kit. Relative expression level for each gene was normalized and calculated using β-actin as the control gene. The amplified PCR product was then subjected to gel electrophoresis for visualizing the DNA bands. RT-PCR analysis experiments were repeated at least two times.

### 4.7. ELISA

To determent the effects of saponins on APP processing and Aβ degrading enzymes, the level of APP proteins and Aβ peptides was measured by ELISA using the mouse amyloid precursor protein ELISA kit and the mouse amyloid beta peptide 1-42 ELISA kit (MyBioSource), respectively, following the manufacturer’s instructions. SweAPP N2a cells were grown in six-well plates and treated with various concentrations of saponin. Cells were harvested and lysate was prepared and processed in 96-well microplates for the experiments. Absorbance was measured at 450 nm using an ELISA microplate reader. Data were analysed and compared following the manufacturer’s instructions. ELISA assay for each protein was performed in triplicate.

### 4.8. Western Blotting

SweAPP N2a cells were treated with saponins. After treatment, cells were washed with PBS buffer and were lysed on ice with radioimmunoprecipitation assay buffer (Sigma-Aldrich). Lysed cells were sonicated and centrifuged and the supernatant containing the proteins was collected. Protein concentration in the supernatant was measured using the BCA protein assay (Pierce). Fifty micrograms of each total protein were separated by 10% sodium dodecyl sulphate polyacrylamide gel electrophoresis. Proteins were transferred from the gel to nitrocellulose membranes (Bio-Rad). The membrane was washed with double distilled H_2_O and blocked in 5% (*w*/*v* non-fat dry milk in Tris-buffered saline (TBS) for 1 h at ambient temperature. Membranes were then hybridized for 2 h at 4 °C with primary antibodies against ADAM10, BACE1, PS1, NCT, (IDE, neprilysin, Aß peptides and APP). TBS containing 5% non-fat dry milk was used for antibody dilutions. Each membrane was washed with double distilled water and incubated for 2 h with secondary antibody conjugated to horseradish peroxidase (1:1000, Pierce Biotechnology) and chemiluminescence was used for visualizing protein bands. Fluor-S MultiImager TM software (Bio-Rad, Hercules, CA, USA) was used for quantification and normalization analysis of the protein bands. Experiments were repeated in duplicate. 

### 4.9. Statistical Analysis

Statistical analysis was performed using GraphPad Prism 8 (version 8.2.0 GraphPad Software Inc., Hercules, CA, USA). Normality and homogeneity of data were determined by Kolmogorov–Smirnov (K–S) test and Levene’s test, respectively. Data are approximately normally distributed with *p*  =  0.162. One-way ANOWA was performed with post hoc analysis by Tukey test for determining the statistical significance between various groups. Significance level α was kept at 0.05. Experiments were performed in triplicate and means were calculated. Data are represented as the mean ± SEM. Data were considered statistically significant at *p* < 0.05.

## 5. Conclusions

In conclusion, we show that theasaponin E1 is natural product able to reduce Aβ amyloid formation and accumulation. However, further study and clinical trials are required for incorporating the green tea seed isolated theasaponin E1 in pharmaceutical formulations.

## Figures and Tables

**Table 1:** Primer sequences used for qPCR.

Primers	Accession NO.	Product Size (bp)	Forward	Reverse
**α-secretase/*ADAM10***	NM_007399	881	5′-GCCAGCCTATCTGTGGAAACGGG-3′	5′-TTAGCGTCGCATGTGTCCCATTTG-3′
**β-secretase/*BACE1***	NM_011792	216	5′-GGATTATGGTGGCCTGAGCA-3′	5′- CACGAGAGGAGACGACGATG -3′
**γ-secretase/*PSP1***	NM_001362271	195	5’-CTGTCCAGCTGCTCCAATGA-3’′	5’-CTTGAGTTACTCTGGGGCCG-3’
**γ-secretase/** ***NCT***	NM_021607	186	5′- CTAGAGAGGCCGCTACGAGT-3′	5′- CTCCACTGAGTTTCCCCCAC -3′
**Insulin-degrading enzyme/*IDE***	NM_031156	576	5′-ACCTGTGAACTGAGCTGCAGAA-3′	5′-TAGAAACGTCTGGTCATCCAACA-3′
**Neprilysin**	NM_008604	250	5’-GCAGCCTCAGCCGAAACTAC-3’	5’-TCAGCATCCATCCAAGTAAG-3
**β-actin/*Actb***	NM_007393	172	5’-AGGGAAATCGTGCGTGACAT-3’	5’-GGAAAAGAGCCTCAGGGCAT-3’

**Table 2:** List of antibodies used for Western blotting.

Antibody	Host	Species Reactivity	Target	Source
Anti-ADAM10 antibody	Rabbit	Mouse, Human	ADAM10	Abcam ab1997
Anti-human/mouse BACE1	Monoclonal Mouse	Human, Mouse	BACE1	R&D Systems MAB931
Anti-Presenilin [APS 11] antibody	Rabbit	Mouse, Human	PS1	Abcam ab15456
Anti-NCSTN Antibody	Rabbit	Mouse, Human	NCT	US Biological 364323
Anti-insulin degrading enzyme/IDE antibody	Rabbit	Mouse, Human	IDE	Abcam, ab32216
Human/Mouse Neprilysin/CD10 Antibody	Monoclonal Rat	Mouse, Human	NEP	R&D Systems MAB1126
Anti-amyloid precursor protein antibody	Rabbit	Mouse, Human	APP	Abcam ab2072

## Supplementary Materials

The supplementary materials are available online.

## Abbreviations

Aβ	amyloid beta
ACh	acetylcholine
AChE	acetylcholinesterase
AD	Alzheimer’s disease
apoE	apolipoprotein E
APP	amyloid precursor protein
ADAM10	A disintegrin and metalloproteinase domain-containing protein 10
BACE1	beta-secretase 1
IDE	insulin-degrading enzymes
LC/TOF/MS	liquid chromatography time-of-flight mass spectrometry
NCT	nicastrin
NFT	neurofibrillary tangles
NMR	nuclear magnetic resonance
PS1	presenilin
SweAPP N2a cells	Swedish amyloid precursor protein variant in neuro2a cells
TLC	thin layer chromatography
